# The evidential role of economic models in legal proceedings

**DOI:** 10.1007/s13194-026-00783-4

**Published:** 2026-10-12

**Authors:** Edoardo Peruzzi

**Affiliations:** Institute of Philosophy, Leibniz University Hannover, Hannover, Germany

**Keywords:** Model evaluation, Scientific models, Legal proceedings, Competition, Model application, Economic models, Evidence

## Abstract

Scientific models are increasingly used as sources of evidence in legal proceedings, particularly in competition cases, in which economic experts apply theoretical and empirical models to provide quantitative insights into the competitive dynamics of specific markets. Despite being routinely presented in courts, however, model-based evidence often has limited influence on judicial outcomes, undermining its intended role in the courtroom. This paper illustrates this phenomenon and then argues that the dismissal of model-based evidence in court is largely driven by what I call models’ large “attack surface”, which makes model-based evidence particularly vulnerable to challenge in adversarial proceedings. I then show that such dismissal is not without consequences. When model-based evidence is excluded, it is typically replaced by market concentration indices, which are more accessible forms of evidence but also less closely related to the target phenomenon. I conclude by arguing that it is epistemically problematic when courts systematically privilege simpler but less target-relevant sources of evidence.

**Supplementary Information:**

The online version contains supplementary material available at https://doi.org/10.1007/s13194-026-00783-4.

## Introduction

Scientific models are routinely employed in various social and institutional contexts, often accompanied by high expectations of enhancing reasoning and strengthening the evidential basis for decision-making. Notable examples recently studied by philosophers of science include the use of epidemiologic models for public health policies (Northcott, 2022; Van Basshuysen & White, 2021; Winsberg & Harvard, 2024), model ensembles in climate science and economics (Gluck, 2023; Jhun, 2023; Parker, 2010, 2011), and natural hazard models in probabilistic risk assessment (Roussos et al., 2021; Zanetti, 2024). A recurring lesson from this literature is that the use of models in policy-relevant settings raises both epistemic and practical challenges, particularly in relation to the aggregation of outputs under conditions of model uncertainty (Jhun, 2023; Roussos et al., 2021; Zanetti, 2024) and the pressures of time-sensitive “fast science” decision-making (Friedman & Šešelja, 2023; Northcott, 2022; Van Basshuysen & White, 2021; Winsberg et al., 2020; Stegenga, forthcoming). So far, however, philosophical attention has largely been confined to scientific and policy contexts, especially cases involving expert disagreement or the use of models in policy evaluation. Yet there is a third, often overlooked domain in which scientific models play a crucial and high-stakes role: the courtroom. This lack of attention is unfortunate, because, as I will argue in this article, the legal setting raises distinctive philosophical and practical challenges that have received comparatively little attention in discussions of scientific modeling.^1^

In this article, I study how models are used in courts of justice, with a focus on the application of economic models in competition cases. Building on this case study, I advance two main claims. First, I argue that model evaluation in legal contexts requires distinguishing between epistemic and legal dimensions of assessment: models that are adequate relative to epistemic standards may nonetheless fail to satisfy the requirements governing their use as legal evidence. In particular, legal adequacy requires that model-based evidence be able to withstand adversarial scrutiny and remain stable under contestation in court. Second, I identify a phenomenon that has received little attention in the philosophy of modeling, namely that model-based evidence may be systematically more vulnerable to dismissal than other forms of evidence, not because it is epistemically inferior, but because the discretionary choices involved in its construction generate a larger “attack surface” in adversarial settings. I further argue that this vulnerability has important consequences for judicial decision-making, since courts are nevertheless required to reach a verdict. Excluding model-based evidence therefore shifts evidential weight onto alternative sources, most notably index-based measures such as market concentration, which are simpler but less relevant to the competitive effects under investigation.^2^

The philosophical significance of this case is twofold. First, it calls for a refinement of adequacy-for-purpose accounts of model evaluation (Parker, 2020), by showing that the concept of “purpose” must be disaggregated into distinct epistemic and legal dimensions when models are used as evidence in legal adjudication. Second, it suggests a more general lesson for the philosophy of modeling: when scientific models are used as evidence, model evaluation cannot be carried out in isolation, but must be understood comparatively, in relation to other evidential resources that may affect decision-making. Studying the use of scientific models in the courtroom thus not only refines existing accounts of model evaluation, but also reveals a distinct and underappreciated pattern in how models function as evidence in practice.

My argument proceeds by introducing, in Section 2, the practice of economic expert testimony in US legal proceedings involving alleged violations of competition law (hereafter “competition cases”). I will show that in the legal context models are introduced by expert witnesses as evidence intended to influence judicial decisions. Courts, in turn, are required to assess the strength and reliability of this model-based evidence when reaching their verdict. Section 3 then turns to a puzzling and systematic phenomenon, namely the recurrent neutralization of model-based evidence in competition cases. Drawing on US case law and the antitrust literature, I show that economic models often fail to exert meaningful influence on judicial outcomes.

Sections 4 and 5 develop the theoretical framework needed to explain and evaluate this phenomenon. Section 4 reviews the adequacy-for-purpose account of model evaluation in the philosophy of science and economics. Section 5 builds on this literature by developing an amended account tailored to courtroom use, based on a distinction between the epistemic and legal purposes that models serve when introduced as sources of evidence. This amendment shows how standard criteria of model adequacy must be revised when models are assessed not only as tools of explanation or prediction, but as inputs to adversarial decision-making. Finally, Section 6 examines the consequences of excluding or marginalizing model-based evidence. It shows that such evidence is often replaced—explicitly or implicitly—by more accessible but less informative forms of evidence, with implications for the evidential basis on which verdicts are reached.

Before proceeding with the main content of this article, a few disclaimers are in order. First, my focus in this article is not on the systemic influence of economic thought on the legal system, but rather on specific ways in which economic models impacts court verdicts in competition cases. Second, I will set aside the long-standing debate on the goals of competition policy and proceed on the assumption that courts enforcing antitrust laws aim to protect competition and consumers – whatever that may entail in specific circumstances (see, e.g., Esposito, 2022; Giocoli, 2024; Martin, 2007; Schinkel, 2008). Finally, given my focus on US legal system, the terms “antitrust”, “competition law”, and “competition policy” will be used interchangeably.^3^ Also, I follow the common practice in US legal scholarship and use “courts” and “judges” as synonyms.

## Model-based evidence in competition cases

Our case study concerns the use of economic models in competition cases, that is, legal disputes concerning alleged violations of antitrust laws, such as monopolistic practices, anti-competitive agreements, and abuse of market dominance, among others. In these cases, economists are often hired as expert witnesses to construct models of counterfactual scenarios, that is, of what would have occurred in the absence of the anticompetitive conduct.^4^ Antitrust practitioners usually refer to this counterfactual scenario as the “but-for” world to indicate the world that one should expect to exist but for the alleged anticompetitive behavior. Ideally, the comparison between the actual world and the but-for world demonstrates the effect of the antitrust violation.^5^

An important example of but-for modeling is merger simulation models, a family of models used in applied economics to predict the effects that a merger between two or more firms will have on the relevant market and its level of competition. The intuition behind their use is simple. In markets that are already concentrated or contain only a few firms, a merger can reduce competition and harm consumers. This can happen in different ways. For example, firms may find it easier to coordinate and raise prices when there are fewer competitors. In addition, even without any coordination, the merged firm may be able to profitably raise prices on its own because it no longer competes with the firm it has acquired and can retain some of the customers who would otherwise have switched between them. At the same time, mergers may generate efficiencies, such as cost savings, that can potentially be passed on to consumers. Predicting the overall effect of a merger is therefore a complex exercise: without a model that compares these various effects using data from the specific market, it is difficult to foresee whether a merger will benefit or harm consumers. Merger simulation models are particularly helpful because they allow economic experts to quantify these competing effects in a systematic way. As a result, they play a central role in merger reviews, that is, court-based assessments of the competitive effects of mergers.^6^  

In general, merger simulation models integrate theoretical and empirical methods within a four-step procedure.^7^ First, an appropriate oligopoly model is selected to represent how firms are assumed to interact in the market, with Bertrand and Cournot competition models being the most common choices. The Cournot model is a standard workhorse of industrial economics. It describes an oligopoly market in which firms produce a homogeneous product at constant marginal cost and compete by choosing the quantity of output they produce. Its price-setting analogue is the Bertrand model, where firms compete by selecting prices instead of quantities.^8^ Second, the most suitable functional form for the market demand system is chosen in order to represent consumer behavior – commonly a logit or nested-logit specification, although more flexible forms are sometimes used. Third, the parameters of the chosen demand system are calibrated or estimated using pre-merger data from the actual market. This step connects the theoretical structure to empirical reality because it ensures that the model reproduces observed market outcomes, such as prices and market shares, before the merger. Finally, the full simulation is performed. This requires solving the calibrated model under the post-merger ownership structure to compute the new equilibrium, which typically includes predicted prices, quantities, and sometimes measures of consumer welfare.

More generally, the example of merger simulation models illustrates the main epistemic role that economic models play in competition cases, namely as sources of evidence. As a working definition, I call *model-based evidence* any item or piece of information derived from scientific models that can influence what one is justified in believing about a particular state of affairs.^9^ For instance, if a merger simulation model predicts that a proposed merger will result in a 5 percent price increase, this prediction becomes part of the model-based evidence presented in the case. Although this paper focuses primarily on economics, comparable forms of model-based evidence appear across many scientific domains routinely invoked in legal proceedings. Perhaps the most prominent example is DNA analysis, which relies heavily on statistical and probabilistic modeling. Such models estimate the likelihood that DNA recovered from a crime scene matches a suspect, often providing decisive evidence in criminal trials.^10^

It is important to distinguish model-based evidence from other types of evidence that can also play a significant role in legal proceedings, especially in competition cases One important category is what I will, for lack of a better term, refer to as *index-based evidence*. Index-based evidence consists of relatively simple quantitative indicators of market structure based on observable data, such as market shares and measures of concentration.^11^ These indexes do not directly track the level of competition in markets, but rather summarize how concentrated a market is in a concise way, without relying on an explicit economic model. As I will discuss in detail below, the role that models play in legal proceedings can only be properly assessed not in isolation, but in relation to other types of evidence that may, in practice, replace them as the decisive basis for judicial verdicts.

## The neutralization of model-based evidence

A notable phenomenon observed by competition scholars and legal authorities is that, despite being routinely presented in courts, model-based evidence is often “neutralized” during legal proceedings, thereby diminishing or even nullifying its impact on judicial outcomes. To better understand this phenomenon, it is useful to examine in more detail the institutional context in which such evidence is presented and evaluated.

In the Anglo-American common law system, which is the focus of my analysis, disputes are resolved through the adversarial system, where parties present their cases in a public forum before independent judges.^12^ Each party hires its own lawyers and, potentially, scientific experts to present a partisan view of both the law and the facts. The goal of the adversarial system is, in principle, to reveal the procedural “truth” through the clash of opposing, self-interested views, presented on equal footing.^13^ In such a context, it is standard practice for the plaintiff’s expert to present the results of a model, while the defendant responds with detailed critiques of the modeling choices made by the plaintiff’s expert, sometimes even offering an alternative model. The key phenomenon I want to highlight is that, in such cases, judges often refrain from taking a definitive stance on this evidence and instead effectively set it aside. In this way, model-based evidence ultimately plays little or no role in the court’s verdict.

The 2020 *Peabody* case provides a relevant example of the neutralization of model-based evidence. In this case, the Federal Trade Commission (FTC) sought to block a proposed joint venture between two coal mining companies, Peabody Energy and Arch Resources, arguing that it would severely harm competition in a region supplying 40 percent of America’s coal.^14^ The court had to make a decision on the hypothesis that the merger would significantly reduce competition (understood in terms of higher prices paid by consumers after the merger), thereby violating antitrust laws. To reach such a decision, the court also needed to assess, alongside other types of evidence, the model-based evidence presented by the expert witnesses of the opposing parties. The FTC presented the expert opinion of Dr. Nicholas Hill, who worked for the leading economic consulting group Bates White. The court was initially swayed by the market definition presented by the FTC’s economic expert, who effectively argued for a narrow relevant market consisting of coal sourced from the Powder River Basin.^15^

With the relevant antitrust market established, the FTC needed to provide evidence that the merger would generate anticompetitive effects. As a first step, the plaintiff’s expert relied on index-based evidence, pointing to a significant change in the Herfindahl–Hirschman Index (HHI) post-merger. The Herfindahl–Hirschman Index (HHI) is a common measure of market concentration that varies from 0 to 10,000. It is calculated squaring the market share of each firm competing in the market and then summing the resulting number. Dr. Hill calculated that the two companies had a combined market share of 68 percent as measured by production volumes. Moreover, he computed the HHI using 2019 mine production data and found that the merger would increase the HHI by 2258 points, from 2707 to 4965.^16^

Eventually, to provide further evidence to support its case, the FTC’s expert employed a merger simulation based on the Cournot model, a well-known model from industrial economics that represents an oligopoly in which firms producing a homogeneous product with constant marginal costs compete by selecting output quantities. Accordingly, the plaintiff’s expert modeled the market as one in which a few coals mining companies (including the two merging companies, Peabody Energy and Arch Resources) compete with each other, offering similar products and having similar cost structures. Applying the Cournot model using available data on prices, quantity, and demand substitution patterns, the plaintiff’s expert argued that if the merger were allowed, it would cause “significantly higher prices for SPRB [Southern Powder River Basin] coal over the next ten years, causing total harm with a net present value of over $1 billion.” (ibid., 906). The plaintiff argued that the model results constituted solid evidence that “the parties’ [...] claimed marginal cost efficiencies” would not prevent the joint venture from “substantially reducing competition, raising prices, and harming consumers.” (ivi).

The defendants, supported by their own economic expert, Dr. Mark Israel, challenged the plaintiff’s use of the Cournot model. Their main objection was that Dr. Hill’s model did not incorporate certain dynamic factors likely to affect how the merger would impact consumers over time. In simple terms, incorporating such factors means taking into account how market conditions may evolve, rather than assuming that current patterns of demand and competition will remain unchanged. In particular, the defendants argued that the model failed to reflect the ongoing transition in the energy sector. Given the rapid growth of renewable energy in recent years, demand for coal is expected to decline as consumers switch to cleaner alternatives. The defendants’ expert pointed to evidence that the number of coal-fired power plants has been steadily decreasing and is likely to continue doing so. In this context, coal producers face an important constraint: raising prices today could accelerate plant closures and lead to a permanent loss of demand to natural gas and renewables. Because Dr. Hill’s model did not account for these factors, the defendants argued, it effectively treated demand for coal as more stable than it actually is. As a result, the plaintiff’s economic model was likely to overestimate the harm caused by the merger, since it ignored the external pressures that would limit the merged firm’s ability to raise prices. More broadly, their critique was that the plaintiff’s model did not adequately reflect some relevant characteristics of the coal industry and therefore could not provide sufficiently reliable evidence to guide the court’s decision.

The court discussed the plaintiff’s merger simulation model and the critiques raised by the defendant over several pages, including suggestions from the defendant’s expert for variations in the plaintiff’s model that resulted in opposing price predictions. However, in its written verdict, the court decisively stated that it did not take a position on the model-based evidence, that is, the evidence resulting from the application of economic models – in this case, to predict the merger’s effect on prices. “This Court,” we read, “need not decisively sift through various models and theories.” (ibid., 907) Thus, model-based evidence did not play a role in the court’s verdict; it was irrelevant as it did not alter the judge’s hypothesis about the competitive effects of the merger.

*Peabody* is not an isolated case; similar expressions regarding the irrelevance of the evidence produced by economic models can be found in various cases litigated before US federal courts. For instance, in *Tronox* (2018), after evaluating the empirical models proposed by competing experts, Judge McFadden remarked:

While the Court found them [empirical models] ultimately consistent with the other evidence presented by the plaintiff’s economic expert, his [model-based] analysis was not dispositive on either the relevant market or the likelihood that the merger will increase market concentration.^17^

The court’s reluctance in *Tronox* reflects a broader trend in antitrust litigation, in which judges often avoid choosing between the economic models presented by the plaintiff and the criticisms raised by the defendants. In fact, the courts in both *Peabody* and *Tronox* explicitly relied on a legal precedent established in *Sysco* (2013), which determined that courts should not decide which economic model is most appropriate when ruling on a competition case.^18^

This tendency of antitrust courts to overlook or neutralize model-based evidence has not gone unnoticed by legal scholars, legislators, and economists. The ensuing debate often focuses on whether, given their limited impact on judicial decisions, the use of economic models in court is justified at all. If model-based evidence systematically fails to influence verdicts, it is natural to question whether the considerable costs associated with its production and assessment are outweighed by its benefits. To give an extreme example of this concern, Jonathan Kanter, who served as Assistant Attorney General at the Department of Justice Antitrust Division during the Biden administration, has voiced strong skepticism about the role of economic models in legal proceedings:

We spend millions arguing about models of the economy and how conduct will hypothetically shift outcomes to the fourth decimal point up or down. Plaintiffs and defendants offer experts to present quantitative models. More often than not, courts reject both competing models and do not believe either side.^19^

Settling the ongoing debate among antitrust practitioners and competition economists is beyond the scope of this article.^20^ My aim in this section is to show, on the basis of case law and the surrounding public debate, that model-based evidence often fails to meaningfully influence court verdicts. Once this is the case, however, courts still face the institutional requirement to reach a decision. This means that the dismissal of model-based evidence must be accompanied by reliance on other available forms of evidence, most often what I have called index-based evidence.

To see how this substitution works in practice, consider again the *Peabody* case, in which the court ultimately set aside model-based evidence after competing expert testimony. Instead, the decision was driven primarily by simpler forms of evidence, in particular evidence concerning changes in market share and market concentration following the proposed merger. More specifically, the court argued that “the FTC’s HHI analysis created a ‘presumption […] that [the Joint Venture] will substantially lessen competition by showing that the JV [Joint Venture] will result in significant market share and an undue increase in concentration’ in the SPRB [Southern Powder River Basin] coal market” (*Peabody*, p. 907). This pattern can also be observed in many other recent judicial opinions on antitrust violations, where model-based evidence is set aside and index-based evidence appears to be decisive for the court’s decision (see, e.g., Budzinski & Noskova, 2022; Rafkin & Kuykendall, 2021).

The dismissal of model-based evidence in favor of index-based evidence that we observe in many competition cases calls for both a descriptive and a normative question. First, why does the neutralization of model-based evidence so often coincide with the decisive reliance on alternative evidentiary forms, in particular index-based evidence? Second, are courts justified in treating model-based evidence in this way, or does its systematic exclusion risk disregarding reliable scientific input that ought to inform judicial decision-making?

Answering these questions requires engaging with a more fundamental issue in the philosophy of scientific modeling: under what conditions can model-based evidence be considered sufficiently reliable to guide judicial decisions? A standard way of addressing this question is to rely on accounts of model evaluation in the philosophy of science, that is, accounts that specify criteria for assessing model quality and clarify how such assessments should be made depending on the context of use. In what follows, I first develop an account of model evaluation tailored to the use of models in the courtroom. More precisely, I depart from the adequacy-for-purpose view, which I refine in light of the institutional and epistemic constraints of legal adjudication. I then return to the comparison between model-based and index-based evidence, emphasizing that model evaluation in practice cannot be carried out in isolation from the other evidential resources that courts treat as dispositive in reaching verdicts. This allows for a more precise characterization of the relationship between these two forms of evidence and, ultimately, for a reassessment of the epistemic role that model-based reasoning should play in judicial decision-making.

## Model evaluation in legal proceedings

One popular way of thinking about model evaluation in science and policy is to check the adequacy of models for a given purpose. Such an adequacy-for-purpose (henceforth AFP) view, which has been recently articulated by Wendy Parker (2020), finds its roots in the understanding of scientific models as tools which should not be evaluated solely for their representational accuracy with respect to a target system but rather for how well they serve certain purposes (e.g., Morgan & Morrison, 1999). In the adequacy-for-purpose view, models are not assessed solely in terms of their truth or representational fidelity. Instead, the evaluation happens via adequacy-for-purpose hypotheses, which specify whether the model is apt for the particular task at hand. For example, an AFP hypothesis might take these forms: “Model M is adequate for the purpose of exploring the implications of imposing a policy of price controls on the cigarette industry,” or “Model M is adequate for the purpose of finding out whether the exposure to asbestos causes long-term health effects.”

One important advantage of the AFP view is that it makes particularly salient that, when assessing a model’s adequacy, one must consider not only the model’s fidelity to the target system, but also features of the user, their ways of employing the model, and the background conditions in which they operate. Parker characterizes the problem space to which a model is a solution as constrained by the target T, user U, methodology W, circumstances C, and goal P. Accordingly, for a model to be adequate for a given purpose, “it must stand in a suitable relationship not just with a representational target T, but with a target T, user U, methodology W, circumstances C and goal P jointly” (Parker, 2020, p. 464). An implication of the AFP view, which I will discuss below, is that a model can fail to be adequate not only because it falls short along some dimension of representational fidelity (T), but also because it fails to satisfy any of the other constraints (e.g., U, W, C, and P) that jointly define the evaluative context.

Interestingly, a closely related line of work in the philosophy of economics has developed similar ideas about model evaluation in a more discipline-specific context. A central and long-standing problem in the field concerns how to assess whether an economic model adequately applies to real-world economic systems (e.g., Aydinonat, 2018; Gibbard & Varian, 1978; Hausman, 1992; Hoover, 2023; Jhun, 2021; Mäki, 2018). Although some approaches already embed model evaluation in a pragmatic context that incorporates the modeller’s purposes and intended audiences (e.g., Mäki, 2009), Rodrik’s *Economics Rules* (2015) offers a particularly influential and practitioner-oriented development of this idea.

Rodrik starts from the familiar criticism that economic models rely on unrealistic or false assumptions and therefore cannot be straightforwardly applied for explanation or policy intervention. He rejects this conclusion, arguing instead that model applicability depends not only on overall resemblance to the target system, but also on the purpose of the model, the context of its use, and the intended audience. These factors help determine which assumptions matter in a given application. As he puts it, “what matters to the empirical relevance of a model is the realism of its critical assumptions” (Rodrik, 2015, p. 55). For example, suppose an economist applies the perfectly competitive model to assess the effects of cigarette price controls. In this context, the assumption that firms lack market power is critical, since relaxing it would substantially change the predicted effects of the policy. By contrast, other features of the model, such as the exact number of firms or finer details of rationality, are less consequential for this application and therefore not critical. Rodrik’s contribution also revitalised the debate on model selection, understood as the procedure of choosing one or more models from a broader model “library” for a specific target and purpose. The key link to critical assumptions is that candidate models are distinguished and selected on the basis of how their critical assumptions perform in relation to the intended application (e.g., Grüne-Yanoff & Marchionni, 2018; Peruzzi, 2023).

Although these considerations are developed in the context of scientific modeling more generally, they raise an immediate question which applies to the courtroom context: how should adequacy-for-purpose be assessed when models are used as sources of evidence in legal proceedings? In particular, what standards should guide the evaluation of whether a model is sufficiently adequate for their evidential role? Parker leaves open how the evaluation of adequacy-for-purpose does or could proceed in practice in different scientific contexts. In what follows, I characterize such standards for the legal context, suggesting that in court proceedings two dimensions are especially important for the evaluator: model selection and model performance.

The first step for the evaluator is to assess whether a specific theoretical model has been appropriately selected from a wider library of available models given the intended application. This is obviously important because economists can choose between multiple models for a given phenomenon – e.g., the effect of mergers on consumers’ prices. This abundance of models, though, raises a well-known methodological challenge: what criteria should guide the selection of the most appropriate model for a given application? This assessment can be carried out in different ways. As we have seen, a philosophically informed approach is to distinguish and select candidate models on the basis of their critical assumptions (Grüne-Yanoff & Marchionni, 2018; Peruzzi, 2023; Rodrik, 2015).^21^ On this view, the evaluator assesses whether an appropriate theoretical model was selected by asking whether these assumptions are sufficiently realistic given the intended purpose. For example, in predicting the effects of price controls in the cigarette industry, a perfectly competitive model may be selected if, among other things, its assumption about firms’ market power is adequately supported by the evidence in that context. This way of reasoning extends naturally to merger simulation models. If an evaluator learns that, in a given case, firms’ products are not close substitutes and that capacity constraints are negligible, she can rule out models based on product homogeneity or quantity competition as inadequate for predicting post-merger price effects. Conversely, the same information supports selecting a differentiated-products Bertrand merger simulation model, insofar as its critical assumptions (price competition and product differentiation) are sufficiently aligned with the characteristics of the relevant market.

The second step concerns model performance. Here, the evaluator may ask what can be learned from the model’s outputs, given the specifics of the legal context. A distinctive feature of models used in court proceedings is that they are often employed to analyze counterfactual scenarios—such as post-merger or but-for price levels—that are not and may never be observed. As a result, direct testing of model performance is typically impossible. For this reason, the evaluation of model performance in legal proceedings must rely on indirect forms of assessment (see also Peruzzi, 2023, Sect. 4). One source is historical evidence: where possible, the evaluator can examine whether the model reproduces observed features of the actual market, such as price levels or trends over a relevant period. For instance, merger simulation models are often expected to match average pre-merger prices in the industry, and failure to do so would count against their adequacy for the purpose at hand (Werden et al., 2004). A second source is indirect evidence from comparable contexts. When the target of the model is a counterfactual scenario for which no historical data exist, the evaluator may consider whether the model’s predictions are broadly consistent with evidence from similar markets or related industries. For example, if a merger simulation model predicts but-for prices for apples at around 10 dollars, and prices in comparable fruit markets typically range between 8 and 13 dollars, this may support treating the model’s output as sufficiently plausible for the purpose at hand. Eventually, economic experts may use sensitivity analysis to show how alternative choices of functional forms and parameters influence the result of the empirical model.^22^ A typical example consists in estimating elasticities of demand from the same body of data but using a variety of different assumptions about the functional form for the demand system and seeing how price predictions respond to the change of values for the elasticities of demand.

Does the adequacy-for-purpose (AFP) view help us to understand why model-based evidence is often disregarded by courts? Prima facie, it does. Recall that, on the AFP view, a model is adequate only if it stands in a suitable relationship not just to its representational target, but also to a user, a methodology, a set of circumstances, and a goal. From this perspective, the neutralization of model-based evidence can be interpreted as a case of misalignment within this broader problem space. In particular, one might argue that the model is inadequate for the legal purpose because its features fail to align with those of its users, namely, the fact-finders (judges or jurors). This possibility is explicitly considered by Parker (2020, p. 465), who notes that a model may be inadequate when, although its equations are highly accurate, they are “so complex that explanatory information is not salient to users.” Applied to our case study, this would mean that the results of a merger simulation model may be epistemically accessible to trained economists but not to legally relevant users such as lay jurors or generalist judges. In such cases, the failure of model-based evidence to influence the verdict can be understood as a failure of adequacy-for-purpose: the model is not adequate with respect the epistemic capacities of its intended users.

Although a diagnosis of the neutralization of model-based evidence on the basis of the AFP view is plausible, it does not take us very far. At best, it identifies a possible locus of misalignment within the problem space, but it leaves open what kind of misalignment is doing the explanatory work in legal contexts. In particular, an evaluation of models based on the AFP view does not explain why epistemically well-supported models may nonetheless be systematically filtered out in adversarial proceedings. Moreover, if taken at face value, this line of reasoning risks collapsing the phenomenon into a simple verdict of inadequacy-for-purpose. Yet, as Parker herself acknowledges, the AFP framework does not specify where “responsibility” for such model inadequacy lies (Parker, 2020, p. 474). It therefore remains indeterminate whether the source of failure is the model, the users, or the institutional setting in which evaluation occurs.

For these reasons, while the AFP view provides a useful starting point, it is nevertheless too coarse-grained to fully explain the fate of model-based evidence in legal proceedings. In the next section, I will extend the AFP framework to address this explanatory gap. I argue that we need to distinguish between epistemic and legal purposes, and that model-based evidence is characterized by a large “attack surface,” which helps explain why epistemic adequacy does not reliably translate into persuasive force in legal proceedings.

## Why is model-based evidence neutralized?

As an amendment to the AFP view, I suggest we distinguish between two distinct purposes for which economic models are employed in legal proceedings. First, models serve an *epistemic* purpose. This consists in generating knowledge about a counterfactual scenario, such as what would have happened in the absence of certain anticompetitive behavior or whether a merger would have led to price increases. Second, models serve a *legal* purpose, namely to provide evidence aimed at persuading the fact-finders of the plausibility of a given hypothesis. These two purposes are conceptually distinct, even if they are often intertwined in practice. The epistemic purpose concerns the production of accurate representation about counterfactual states of the world, whereas the legal purpose concerns the role of models as forms of legal evidence, in particular their capacity to shape what fact-finders are justified in believing in a legal setting. On this view, the function of economic models is not limited to generating accurate representations of economic reality, but also extends to influencing judicial decision-making—for instance, by supporting the conclusion that a merger is likely to harm consumers through higher prices.^23^

Distinguishing between an epistemic and a legal purpose has an immediate consequence: it allows us to avoid treating models as simply *tout court* adequate or inadequate. Instead, adequacy must always be assessed relative to a specific purpose. This is not in tension with the AFP framework; rather, it provides a more fine-grained specification of how that framework should be applied in the legal context. Once we recognize that economic models in litigation serve both an epistemic and a legal purpose, it becomes possible to explain a range of cases in which models appear to “fail” without concluding that they are defective as models. Such an explanation relies on the fact that no relation of logical dependence exists between the two purposes. In particular, it is perfectly possible for a model to be adequate for the epistemic purpose while still failing to be adequate for the legal one. A model may, for instance, be appropriately chosen from the available set of competing models given the epistemic task, in the sense that its assumptions are sufficiently well supported with respect to the phenomenon of interest. Yet it may still fail to persuade the court because those assumptions are not sufficiently transparent to fact-finders or because opposing experts successfully cast doubt on key modeling choices made by the expert witness.^24^ This distinction therefore offers a more precise diagnosis of the neutralization of model-based evidence. It separates cases in which a model is epistemically inadequate from cases in which it is epistemically appropriate but nevertheless fails to perform its legal function.

In practice, the distinction helps us reinterpret cases such as *Peabody*, as well as many other instances in which economic models do not ultimately influence judicial decisions. On the present account, such cases need not be understood as showing that the models are inadequate simpliciter. Rather, they can be reconstructed as cases in which a model may be adequate for its epistemic purpose—providing a coherent and methodologically sound account of a counterfactual scenario—while failing to achieve its legal purpose of persuading the court. This failure may stem from a variety of factors: the model’s assumptions may be contested by opposing experts, its results may be presented in a way that lacks transparency, or the court may require a higher standard of robustness or intelligibility than the model provides.

Once we distinguish between the two purposes, a further question arises: what explains the fact that epistemically adequate models nonetheless often fail to fulfil their legal function? To address this, I introduce the notion of an “attack surface.” This concept captures the idea that model-based evidence is particularly vulnerable to challenge in adversarial legal settings, even when it is methodologically sound. More specifically, I argue that economic models in litigation typically exhibit a large attack surface, meaning that they contain multiple points at which they can be plausibly challenged by opposing experts. This vulnerability is not incidental but stems from a structural feature of economic models as used in legal proceedings: their high degree of modeling freedom, that is, the wide range of modeling choices available to the modeller. This flexibility creates ample room for critique in adversarial contexts and can even enable the construction of multiple competing models, the evaluation of which often exceeds the expertise of even the most well-intentioned judges.^25^

The notion of attack surface is meant to capture two elements. The first is an operational element, which views the attack surface as a feature of how a given piece of evidence is produced. On this view, the more modeling choices are available, and thus open to challenge by the opposing party, the broader the attack surface. In this sense, model-based evidence typically has a wider attack surface than other types of evidence, precisely because more discretionary choices are involved in its production. The second element is rhetorical, that is, about how evidence is perceived in adversarial contexts, independently of its intrinsic strength or reliability. From this perspective, some forms of evidence may be more easily problematized or undermined in the eyes of fact-finders. This is particularly important in adversarial contexts, where persuasive force—rather than epistemic merit alone—often determines which arguments prevail. It is thus a central feature of the attack surface concept that it encompasses both operational and rhetorical aspects, each of which plays a distinct role in the use of scientific evidence in legal proceedings. Indeed, it is both of these aspects that help explain why, in practice, model-based evidence is often set aside in legal proceedings.

**Fig. 1:**
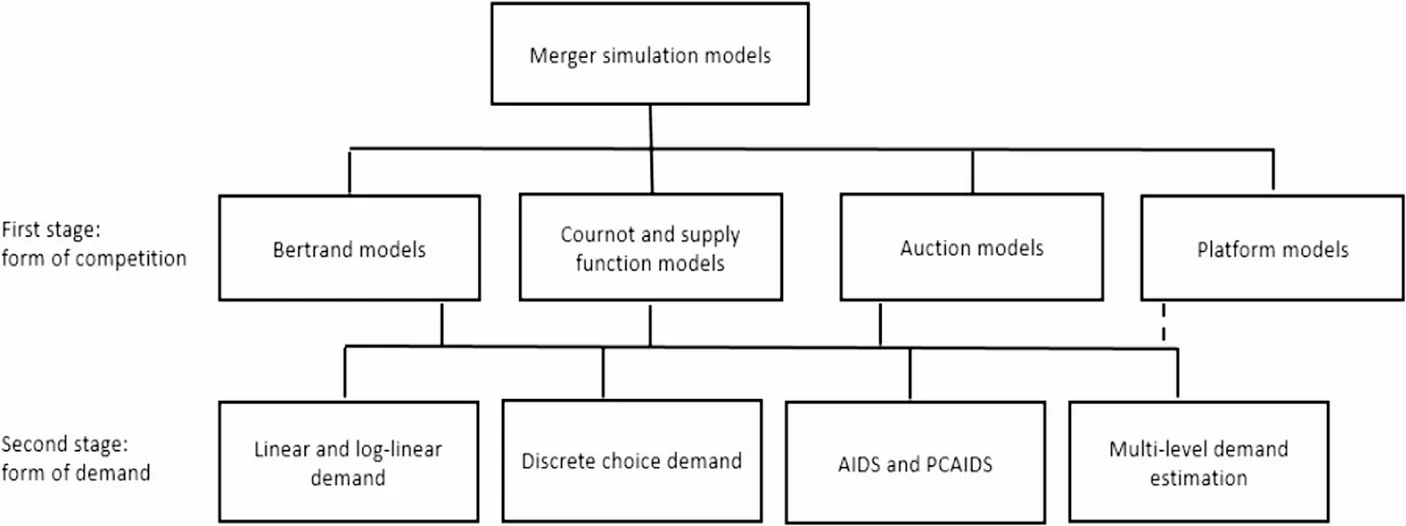
Main components of a merger simulation model, including alternative theoretical models of competition and different specifications of consumer demand. Reproduced from Budzinski and Noskova (2022, p. 59)

Consider, once again, merger simulation models used by expert witnesses to predict post-merger price effects. As we saw, such models require, at a minimum, (i) selecting a theoretical model to represent how competition works in the relevant market and (ii) specifying a demand system which captures how consumers substitute between products in response to price changes (see Fig. 1). Each of these steps involves a set of non-trivial and contestable choices that are typically underdetermined by available theory and data (see, e.g., Panhans & Taragin, 2023). Even if the theoretical model is appropriate for the market in question, further choices are needed to make the model operational for deriving empirical results. Simpler functional forms impose stronger assumptions about product substitution, but they require less data to be estimated and are easier for judges to interpret. Conversely, more complex functional forms reduce unrealistic assumptions but demand more data and are harder to analyze (for an overview of the trade-off in demand specification, see Asker & Nocke, 2021; Budzinski & Ruhmer, 2010; Miller & Sheu, 2021).^26^ As a result, model construction inevitably involves a series of discretionary decisions, each of which can be questioned in adversarial proceedings.

The point is not that these choices are arbitrary or unjustified, that is, that economists lack good reasons for selecting a specific competition model or a functional form for demand systems. On the contrary, these choices are typically well justified (e.g., in the *Peabody* case, coal is homogeneous, making Cournot competition appropriate, whereas in markets with differentiated products, a Bertrand model is more suitable). The problem is rather that model selection often depends on qualitative features of the market whose relevance for model choice remains open to reasonable disagreement and is therefore easily contestable by the opposing party in a trial setting. Once a model is selected, further discretion arises in how such model is operationalized to obtain empirical results, where different but plausible options are available and the “correct” choice is often underdetermined. These alternatives can generate distinct models or competing interpretations of the same data. This is precisely what enlarges the attack surface of model-based evidence: the more degrees of freedom a model contains, the more entry points exist for contestation, reinterpretation, and methodological challenge. Importantly, this is not merely a matter of there being a uniquely correct set of modeling choices that we do not know or cannot identify in advance. Rather, the available evidence and the aims of the analysis may not point to a single choice even in principle: more than one model may be equally consistent with the evidence and suitable for the purpose of the analysis.

That model-based evidence has a large attack surface bears direct implications for how successfully models serve their legal purpose. Even when a model performs adequately from an epistemic perspective—namely, by providing a coherent and well-supported representation of a counterfactual scenario—it may still fail to achieve its legal purpose. The reason is that legal adequacy is not exhausted by truth-conduciveness or explanatory accuracy; it also depends on whether the model can withstand adversarial scrutiny and remain stable under contestation in the courtroom. A model with a large attack surface may therefore be epistemically adequate while remaining legally fragile, because the steps through which model results are obtained are open to contestation that can undermine its persuasive force.

A comparison with index-based evidence is useful here. Although, as I will explain below, index-based evidence relates only indirectly to the phenomenon of market competition, it is simpler to understand and has a smaller attack surface. This is because, once market boundaries are defined, market concentration measures are merely arithmetic calculations. As a result, they are harder to criticize and attack than model-based evidence. The *Peabody* case illustrates this distinction well. Index-based evidence was contested by the defendants, who argued that “this Court cannot simply assume, based on the FTC’s showing of ‘increased concentration in a narrowly defined market,’ that the JV [Joint Venture] will have anticompetitive effects” (*Peabody*, p. 905). However, once the Court accepted the relevant market definition, calculating market shares and HHI variations became a straightforward computational exercise. Consequently, the defendants had little room to challenge the HHI itself, as it was effectively a mechanical outcome based on agreed market parameters, primarily market size and output production. Notably, the Court emphasized that “tellingly, Defendants’ experts do not contest the accuracy of Dr. Hill’s calculations of market share or market concentration” (ibid., p. 903), without acknowledging that challenging the calculation itself would have been nearly impossible.^27^

To sum up, in this section I have argued that the dismissal of model-based evidence in court is largely driven by what I call models’ large “attack surface”, which makes such evidence particularly vulnerable to challenge in adversarial proceedings. The following section will provide a systematic comparison between model-based and index-based evidence. In particular, I will argue that courts should resist dismissing model-based evidence in favor of exclusive reliance on index-based evidence when reaching their decisions.

## Index-based evidence and model-based evidence

The previous section explained why model-based evidence is often legally fragile: its large attack surface makes it vulnerable to contestation in adversarial proceedings, even when epistemically well founded. At this point, the reader might ask why this is a problem at all – why do courts even need model-based evidence? Some might even go further and argue that the neutralization of model-based evidence is not a flaw, but a feature of the adversarial system. According to this view, the mutual neutralization of competing models during a trial reveals a (sort of) “truth”: that model-based evidence is irrelevant for resolving the specific case at hand.

Against this view, I argue that courts in competition cases should not dismiss model-based evidence without due consideration. Because courts must reach a verdict, excluding model-based evidence does not leave the evidential situation unchanged. It shifts the weight of the decision to whatever other evidence remains available. In practice, as illustrated in *Peabody*, this means that more accessible but less model-dependent forms of index-based evidence become more influential in determining outcomes in antitrust cases. My concern, therefore, is not that model-based evidence should always be decisive, but that its systematic sidelining can distort the evidential basis of judicial decisions in competition cases. 

To clarify what is at stake, it is necessary to compare in a more systematic way model-based evidence with the evidentiary alternative that typically takes its place, namely index-based evidence. In doing so, I will explain why courts should show greater appreciation for the role of models in the courtroom and exercise caution when dismissing model-based evidence in favor of index-based evidence.

Index-based evidence differs from model-based evidence along three dimensions: 1) reliance on scientific models, 2) complexity, and 3) target relevance. In particular, index-based evidence does not rely on explicit economic models, is typically simpler to produce and interpret than model-based evidence, and bears a more indirect relationship to the target phenomenon of competition.^28^

First, index-based evidence does not rely on scientific models but, as the name implies, on simple arithmetic indices. The most important types of index-based evidence employed in competition cases are measures of market concentration, which are derived from observable data such as market shares. For instance, the *n*-firm concentration ratio shows the combined market share of the largest *n* firms.^29^ Another example, which we have already encountered, is the Herfindahl–Hirschman Index (HHI) of a market, which is attained by taking the square of the market share of each firm and then summing the numbers together.^30^

Second, index-based evidence is generally simpler than model-based evidence, as it relies on basic arithmetic calculations that are easy to compute and understand, even for non-experts. For example, the Herfindahl–Hirschman Index (HHI), one of the most commonly used measures of market concentration, is a normalized index that can be calculated once market shares are known. Market share itself is typically defined as the percentage of total sales generated by a firm in a given market. The HHI is then obtained by summing the squared market shares of all firms in the market. In contrast, model-based evidence requires the use of more complex economic models and statistical techniques to generate results that can be used as evidence. These models involve explicit assumptions about market behavior and rely on econometric or simulation-based methods to estimate economic relationships or counterfactual outcomes. As a result, model-based evidence is more analytically demanding and less readily accessible to non-specialists, including judges and jurors.

Third, and most importantly, model-based evidence has greater target relevance than index-based evidence. Model-based evidence is designed to track the phenomenon that is directly at issue in antitrust cases, namely the level of competition in a market. As such, it licenses inferences about whether a given conduct affects competitive conditions. By contrast, index-based evidence tracks a different phenomenon: market concentration. It provides information about how market shares are distributed—i.e., the extent to which a small number of firms account for total sales, production, or capacity—but does not directly represent the competitive process itself. For this reason, index-based evidence bears a more indirect relationship to the target phenomenon of competition and is therefore less target-relevant than model-based evidence.^31^

From a philosophical perspective, the difference can be understood in terms of what the evidence is designed to track. Model-based evidence is constructed to represent the target phenomenon (that is, market competition) and can therefore support inferences about it. Crucially, this is because such models, when appropriately specified, embed assumptions about the mechanisms that determine market outcomes (e.g., demand, supply, and the factors generating them). This allows them, at least in principle, to map from observed data to the underlying competitive process in a way that is sensitive to intervention: they are designed to answer what would happen to competition under alternative market configurations. Index-based evidence, by contrast, tracks another phenomenon, namely, market concentration. Its evidential relevance depends on the assumption that concentration is a reliable indicator of competition—an assumption that is neither conceptually guaranteed nor empirically stable across contexts. As a result, the inferences licensed by index-based evidence are indirect and may become misleading when considered in isolation.^32^

This point becomes particularly clear once we consider the causal status of concentration measures. The Herfindahl–Hirschman Index (HHI), for example, measures market concentration, but concentration is not itself a stable causal proxy for competitive effects. Miller et al. (2022), in a paper authored by twenty-six competition economists, including current and former chief economists at the Federal Trade Commission, emphasize that a regression of HHI on price can establish at most a correlational relationship. Such a regression therefore cannot, by itself, support causal inferences about the effects of mergers on prices or competition, since the same observed association may arise from multiple distinct underlying market structures. In this sense, index-based evidence alone does not provide a reliable basis for counterfactual reasoning in antitrust analysis.^33^

In the economics literature this problem is known as the Demsetz’s critique, with Harold Demsetz famously introducing the concept of “concentration through competition,” arguing that concentration is not necessarily a sign of reduced competitiveness but can instead reflect successful entrepreneurial efforts (Demsetz, 1973, 1982). He noted that “if rivals seek better ways to satisfy buyers or to produce a product, and if one or a few succeed in such endeavors, then the reward for their entrepreneurial effort is likely to be some (short-term) monopoly power, and this may be associated with increased industrial concentration” (1973, p. 3). Demsetz’s critique has become part of the common background knowledge of contemporary industrial organization economics. Economists generally regard concentration measures as, at best, rough indicators of competitive harm rather than direct evidence of it. Consequently, there is broad agreement that index-based evidence should not be relied upon in isolation when assessing antitrust liability (e.g., Berry et al., 2019; Carlton & Israel, 2021; Fisher, 2008; Syverson, 2019).^34^ And yet, in many competition cases, it is precisely this type of evidence that becomes legally salient. 

What is worth emphasizing is that the problem of dismissing model-based evidence runs deeper than simply impairing judicial decision-making. In fact, even if courts were always to reach the correct verdict (e.g., convicting guilty firms and acquitting innocent ones), the epistemological implications of systematically discarding model-based evidence would still remain. This is true for one main reason. Courts that rely primarily on index-based evidence risk creating a systematic mismatch between the target of adjudication and the evidence used to assess it. Antitrust law is ultimately concerned with the effects of firm conduct on competition, yet index-based evidence tracks a different phenomenon, namely market concentration. Model-based evidence, by contrast, is specifically designed to represent the competitive process that characterizes a market and to support counterfactual inferences about how competition would evolve under alternative market conditions. When courts privilege index-based evidence while setting aside model-based evidence, they therefore rely more heavily on evidence that is only *indirectly* connected to the phenomenon under investigation.

An analogy from forensic sciences may help clarify the point. Imagine a court systematically giving greater weight to circumstantial indicators, such as a defendant’s proximity to the crime scene, while discounting more directly probative evidence, such as DNA analysis, simply because the latter requires more technical interpretation or is easier for opposing experts to contest. We would regard this behavior as epistemically troubling because it privileges evidence that is more familiar over evidence that bears more directly on the phenomenon under investigation. Something similar occurs when antitrust courts rely solely on index-based evidence in reaching a verdict despite its weaker connection to the competitive effects at issue, thereby creating an epistemically significant disconnect between the phenomenon antitrust law seeks to assess and the evidence on which courts rely in assessing it.

## Conclusion

Let us recap the main steps of this work. I began by examining the use of economic models as evidence in legal proceedings, showing that the evidential support they generate is often dismissed by fact-finders. This phenomenon relates to a question of central interest in the philosophy of scientific modeling, namely how the adequacy of a model should be evaluated in practice. After reviewing the adequacy-for-purpose view of model evaluation, I proposed an amendment aimed at developing an account of model assessment tailored to the courtroom context. This amendment relies on a distinction between an epistemic and a legal purpose that economic models serve when introduced as evidence in legal proceedings. The resulting diagnosis points to a significant tension between these two aims, insofar as models that are adequate for epistemic purposes may nonetheless be inadequate for legal purposes. Eventually, I provide reasons for why the dismissal of model-based evidence is epistemically concerning: when model-based evidence is dismissed, simpler but less target relevant forms of index-based evidence takes its place, potentially lowering the quality of judicial decisions.

This article represents an initial step toward understanding how scientific models are, and perhaps ought to be, employed in legal proceedings. However, much work remains for philosophers of science in this area. The next step would be to explore whether the present analysis is specific to economic models or whether it applies more broadly. Other scientific models, such as forensic models (e.g., ballistic or bloodstain pattern analysis), psychological models (e.g., risk assessments or mental state evaluations), and financial models (e.g., estimating damages in patent or fraud cases), are also used in the courtroom. Future research could focus on whether fields with stronger consensus on reliable methods of evidence gathering – such as DNA or fingerprint analysis – are less prone to the neutralization by legal decision-makers.

## Electronic Supplementary Material

Below is the link to the electronic supplementary material.

Main components of a merger simulation model, including alternative theoretical models of competition and different specifications of consumer demand. Reproduced from Budzinski and Noskova (2022, p. 59).

## Data Availability

N/A.

## Footnotes

1 To be sure, philosophers have examined how courts should evaluate and manage scientific evidence in judicial proceedings (e.g., Beecher-Monas, 2009; Dwyer, 2008; Fischer & Jukola, 2026; Gebharter & Sikorski, 2026; Gerken, 2022; Haack, 2015; Jasanoff, 1997; Martini, 2015; Miller, 2016; Satta, 2022; Sikorski, 2022). However, this article takes a unique approach by focusing on the evidential role of scientific modeling in judicial proceedings.

2 “Fact-finder” refers to the individual or entity responsible for assessing the facts of the case – the judge in a bench trial or the jury in a jury trial, depending on the type of case.

3 Antitrust law was introduced in North America at the end of the nineteenth century to prevent anticompetitive behavior and promote market competition. In Europe and other jurisdictions, antitrust law is referred to as competition law.

4 On the role of economists as expert witnesses, see the classic articles by Mandel (1999) and Posner (1999) and the recent contributions by Maas & Svorenčik (2017), Chassonnery-Zaigouche (2020), Giocoli (2020), and Peruzzi (2023).

5 The notion of the but-for world, adapted from tort law, carries a specific causal meaning, according to which a factor counts as a cause only if it is a necessary condition for the outcome; see, e.g., Qian (2025), who argues that this standard is often ill-suited to antitrust economic analysis.

6 Merger simulation as a tool for competition policy was introduced in the mid-1990s by Hausman et al. (1994) and Werden and Froeb (1994). Since then, a substantial literature has examined the robustness of these models by varying their underlying assumptions, in particular the functional form of demand and the theoretical model of competition (e.g., Epstein and Rubinfeld, 2002; Huang et al., 2008; Ivaldi & Verboven, 2005; Nevo, 2000; Slade, 2009).

7 My description of the basic functioning of merger simulation models draws on the overviews in Budzinski and Noskova (2022), Budzinski and Ruhmer (2010), and Werden et al. (2004).

8 For a standard textbook treatment of the Cournot and Bertrand models and their main extensions, see Tirole (1988).

9 There is significant debate within the philosophy of science about what constitutes evidence (for an overview, see Kelly, 2016 and Ho, 2021). That said, my definition of model-based evidence follows the standard conception of evidence as something that can make a difference to what one is justified in believing.

10 Similar uses of scientific modeling can be found in other forensic techniques, including ballistic and bloodstain pattern analysis, as well as in psychological assessments, such as evaluations of risk or mental state, and in financial models used to estimate damages in patent or fraud litigation.

11 Model-based and index-based evidence do not exhaust the forms of proof used in legal proceedings. Courts also rely on more conventional evidentiary categories grounded in factual record and documentation. Factual evidence refers to direct observations or testimonies that can be verified, such as witness statements about a company’s practices or sales records showing pricing strategies. Documentary evidence includes written or recorded materials supporting a claim, such as contracts, emails, or reports detailing communications regarding pricing agreements or market strategies.

12 The adversarial system contrasts with the inquisitorial system used in many European countries, characterized by a more active role for the judge in directing the case, making inquiries, calling and examining witnesses, and generally determining the matters the court will decide. There are different types of witnesses, including lay witnesses, who are individuals with personal knowledge of the matter, and expert witnesses, who possess specialized knowledge and are called upon to provide expert testimony within their area of expertise. This article focuses exclusively on the latter.

13 Gordon Tullock famously criticized the common law system on this very point, arguing that the clash of self-interested parties is both informationally inefficient and socially wasteful. See Tullock (2004, 2005a, 2005b), and discussion in Albertazzi et al. (2024), Shughart (2018), and Zywicki (2008).

14 *FTC v. Peabody Energy Corp.,* 492 F. Supp. 3D 865 (E.d. Mo. 2020). (“Peabody”).

15 The Powder River Basin is a geologic structural basin in southeast Montana and northeast Wyoming. Roughly speaking, the smaller the market, the easier it is for the plaintiff to demonstrate potential anticompetitive effects of certain business conduct. As such, persuading the judge of a narrow market definition is a strong starting point for winning a competition case.

16 The HHI is currently used as a signal of potential anticompetitive effects of mergers in the European Commission’s Guidelines on the Assessment of Horizontal Mergers and the US Horizontal Merger Guidelines. In Sect. 6 below, I shall further discuss market concentration measures (what I label “index-based evidence”) in contrast to model-based evidence.

17 *FTC v. Tronox Ltd.,* 332 F.Supp. 3 d 187 (D.D.C. 2018).

18 *FTC v. Sysco Corp*., 113 F.Supp. 3 d 1. (“Sysco”). For another example of the irrelevance of model-based evidence in court verdicts, see the opinion issued by Judge Victor Marrero in New York v. Deutsche Telekom AG, 439 F. Supp. 3 d 179 (S.D.N.Y. 2020). There, he argued that the parties’ efforts were reduced to “imprecise and somewhat suspect aids: competing [sic] crystal balls” (188). “The parties’ costly and conflicting […] economic […] models,” he wrote, “along with the incompatible visions of the competitive future their experts’ shades-of-gray forecasts portrayed, essentially canceled each other out as helpful evidence the Court could comfortably endorse as decidedly affirming one side rather than the other” (ibid., 187).

19 Keynote at the University of Chicago Stigler Center, Chicago, IL, Thursday, April 21, 2022 “Antitrust Enforcement: The Road to Recovery.” Transcript of the speech available here: https://www.justice.gov/opa/speech/ assistant-attorney-general-jonathan-kanter-delivers-keynote-university-chicago-stigler. Accessed on April 15, 2026.

20 Many prominent economists have recently taken part in an active internal debate within the discipline on the advantages and risks of using economic models in the courtroom (e.g., Carlton & Israel, 2021; Katz, 2021; Salop, 2021; Shapiro, 2021a, 2021b).

21 Model selection is understood here as a choice among theoretical models in light of a particular application, for which formal selection criteria are often unavailable (for a recent overview, see Schulz, 2025). A distinct notion of model selection applies to empirical models, where one faces the familiar trade-off between simplicity and fit to the data. For a philosophically informed discussion of such a trade-off in economic modeling, see Ruiz and Schulz (2023).

22 There is disagreement in the literature on whether robustness analysis has epistemic value (such as confirmation of model results). In particular, various authors have raised objections to the epistemic merits of robustness analysis (see, e.g., Harris, 2021; Lisciandra, 2017; Lloyd, 2015; McLoone et al., 2025; Odenbaugh & Alexandrova, 2011; Parker, 2011﻿; Woodward, 2006). For a recent overview of the debate, see Lehtinen (2026) and references therein.

23 The amendment suggested here is in line with Watkins (2024), who proposes a refinement of the AFP view by more precisely specifying the structure and relations among possible purposes, suggesting that research purposes should be understood as nested, with some serving more ultimate aims. The main difference from the proposal developed here is that Watkins explicitly focuses on data evaluation (as opposed to model evaluation) and conceives of a hierarchical structure of research purposes.

24 A clarification is in order regarding the relation between my notion of a “legal purpose” and Parker’s notion of a “practical purpose.” Parker distinguishes between epistemic and practical purposes in model use, arguing that even when models are employed for practical ends, their contribution is typically mediated through epistemic purposes: “it is expected that the model’s serving one or more epistemic purposes will, in the context of a more extended activity, facilitate the achievement of the practical purpose” (Parker, 2020, p. 460). My notion of “legal purpose” differs from Parker’s in that, within her framework, epistemic success is expected to instrumentally support practical success. In litigation, by contrast, this connection is not warranted: a model may be epistemically adequate yet fail to achieve its legal purpose, since legal outcomes also depend on how vulnerable the model’s results are to adversarial challenge and on the persuasive uptake of evidence by fact-finders. Accordingly, the legal purpose cannot be reduced to, nor fully captured in terms of, Parker’s notion of practical purpose.

25 The explanation for the neutralization of model-based evidence provided here, which centers on the notion of attack surface, is not in conflict with other explanations that highlight the judge’s lack of economic expertise. On the contrary, it should be seen as complementary, as the two factors reinforce each other: model-based evidence, which already has a large attack surface, is bound to appear even more inscrutable the less economic expertise the judge possesses. Judicial struggles with scientific evidence are in fact well-documented (see Koehler, 2018; Hans & Saks, 2018, and references therein). One potential solution is increasing the use of court-appointed experts to mediate conflicts between opposing expert testimonies (see Lopatka, 2016; Posner, 1999; Sidak, 2013; Rubinfeld & Cecil, 2018; Peruzzi, 2023).

26 For example, a simple logit demand system implies the independence of irrelevant alternatives property, which imposes restrictive substitution patterns across products. In the automobile market, this would imply that a price increase for a BMW leads to proportional substitution toward all other car brands (e.g., Mercedes, Fiat), rather than stronger substitution toward closer competitors such as Mercedes.

27 As an anonymous reviewer has rightly pointed out, I have left open the question of what would make model-based evidence more or less adequate for legal purposes. A full analysis of this question lies beyond the scope of the present article, but a few remarks are in order. First, I do not take the existence of a large “attack surface” to imply that model-based evidence is *intrinsically* inadequate for legal purposes. How well model-based evidence serves its legal purpose depends not only on the characteristics of the model but also on the rhetorical ability of expert witnesses, the scientific literacy of fact-finders, and so on. Second, I contend that simply improving the epistemic adequacy of models is typically not sufficient to increase their legal adequacy. The scope for improving empirical accuracy, for example, is constrained by the need for experts to simplify their models so that their results remain comprehensible to judges and jurors. Model-based evidence may therefore remain highly vulnerable to adversarial challenge even as its epistemic adequacy improves. A more fruitful way to approach the question, which I leave for future work, is to focus on two underappreciated aspects: first, the kinds of *questions* that models are asked to answer in courts of law, since the choice of question can affect how vulnerable the evidential claim generated by the model is to adversarial challenge, particularly when models are better suited to answering some questions than others; and second, how the legal concept of evidence differs from the scientific concept of evidence, since what counts as adequate evidence in law is generally not exhausted by the standards that govern scientific evidence.

28 The distinction between model-based and index-based evidence does not exhaust the kinds of evidence that scientific experts might employ in principle. For example, economists routinely accept other types of evidence, such as randomized controlled trials (RCTs), quasi-experimental empirical research, and laboratory experiments, in their academic work. However, I do not explicitly consider these types of evidence here, as they are generally not yet central in the context of legal proceedings (Einav & Levin, 2010). Indices can of course be used to measure or represent indirectly many different phenomena; here, I focus specifically on market concentration indices because of their prominent role in competition cases. I thank an anonymous reviewer for pushing me to clarify this point.

29 For example, in a market where the three largest firms, A, B, and C, hold market shares of 50 percent, 30 percent, and 10 percent respectively, the 3-firm concentration ratio would be 90 percent (i.e., 50 percent + 30 percent + 10 percent).

30 I should note at the outset that indexes are not created in a theoretical vacuum – they typically rest on underlying theoretical assumptions, and developing them is not necessarily a model-free exercise. For instance, the GDP deflator can be derived using the Keynesian aggregate demand function. More complex indexes, such as the Chicago Fed’s National Activity Index or the Atlanta Fed’s GDPNow, rely on both theoretical and empirical models to combine multiple indicators or generate filtered estimates (see, e.g., Boumans, 2004, 2015 and Reiss, 2008 for an analysis of the relationship between modeling and indexing in economics). The type of indexes I refer to here, however, are based on informal models or simple hypotheses – such as “the more firms, the more competition” – which differ from the fully-fledged economic models that are used to generate what I call model-based evidence.

31 Although the HHI is sometimes described as functioning like a “barometer”, this analogy is potentially misleading. A barometer reliably tracks a single underlying variable (atmospheric pressure) because the measurement relation is stable and physically grounded. By contrast, the relationship between concentration indices and competition is mediated by heterogeneous causal mechanisms. As a result, identical HHI values may be generated by both highly competitive and weakly competitive market structures. This undermines the idea that the index tracks a single, well-defined underlying quantity.

32 This is a claim about *types* of evidence rather than their contingent performance in particular applications. Model-based evidence can of course be mis-specified, poorly estimated, or based on unrealistic assumptions in specific cases. The point here is instead about what we can generally expect from these evidential forms in virtue of their structure: model-based approaches are, in principle, designed to represent and reason about the target phenomenon, whereas index-based approaches are limited to summarizing proxy variables. For a discussion of how indices can function as indirect representations and as instruments for policy evaluation, see Morgan and Bach (2018) on the Human Development Index.

33 Jhun (2025) provides a detailed account of the metaphysical reasons for which, in general, the relation between the HHI of a market and prices cannot be given a straightforward causal interpretation, as well as the conditions under which the HHI may nonetheless function as an indicator of market competitiveness.

34 It is important to note that the distinction between model-based and index-based evidence simplifies a more complex spectrum. In practice, evidence varies in the degree to which it relies on a theoretical framework, with some types of evidence drawing more heavily on scientific models than others. However, for the purposes of this article, this dichotomy serves as a useful working framework. What is crucial for my argument is merely that contemporary economics suggests that index-based evidence, when considered in isolation, is unreliable for assessing competition and should not be the sole basis for competition policy decisions.
